# Quality of life in people living with HIV in Romania and Spain

**DOI:** 10.1186/s12879-021-06567-w

**Published:** 2021-09-13

**Authors:** Meaghan Kall, Ujué Fresán, Danielle Guy, Graham Brown, Cristina Burgui, Jesús Castilla, Victor Ionel Grecu, Florentina Dumitrescu, Valerie Delpech, Jeffrey V. Lazarus

**Affiliations:** 1 HIV/STI Department, Public Health England, London, NW9 5EQ UK; 2 Instituto de Salud Pública de Navarra-IdiSNA-CIBERESP, Pamplona, Spain; 3 Barcelona Institute for Global Health (ISGlobal), Hospital Clínic, University of Barcelona, S08036 Barcelona, Spain; 4 Centre for Social Impact, University of New South Wales, High Street, Sydney, Australia; 5 Spitalul Clinic de Boli Infectioase si Pneumoftiziologie “Victor Babes” Craiova, 200515 Craiova, Romania

**Keywords:** EQ-5D, Chronic diseases, HIV/AIDS, Health-related quality of life, PozQoL, Romania, Spain

## Abstract

**Background:**

Health-related quality of life (HRQoL) is a crucial component in assessing and addressing the unmet needs of people, especially those with chronic illnesses such as HIV. The aim of the study was to examine and compare the health-related quality of life of people living with HIV in Romania and Spain, compared to the general populations of each country.

**Methods:**

A cross-sectional survey was conducted among adults (≥ 18 years) attending for HIV care in Romania and Spain from October 2019 to March 2020. The survey included two validated HRQoL instruments: a generic instrument, EQ-5D-5L, and an HIV-specific instrument, PozQoL, and questions on socio-demographics, HIV-related characteristics, physical and mental health conditions, and substance use. Multivariable linear regression was used to determine factors associated with HRQoL.

**Results:**

570 people living with HIV responded (170 in Romania and 400 in Spain). The median age was 31 (18–67) in Romania and 52 (19–83) in Spain. Anxiety/depression symptoms were frequently reported by people with HIV (Romania: 50% vs 30% in the Romanian population; Spain: 38% vs 15% in Spanish population). Spain reported higher mean EQ-5D_utility_ scores than Romania (0.88 and 0.85, respectively) but identical PozQoL scores (3.5, on a scale of 0–5). In both countries, health concerns were highlighted as a key issue for people with HIV. In multivariable analysis, two factors were consistently associated with worse HRQoL in people with HIV: bad or very bad self-rated health status and presence of a mental health condition. In Romania, being gay/bisexual and being disabled/unemployed were associated with worse HRQoL. Whereas in Spain, older age and financial insecurity were significant predictors.

**Conclusions:**

Our results indicated a good HRQoL for people living with HIV in Romania and Spain; however, worse HRQoL profiles were characterized by health concerns, poor self-rated health status, and the presence of mental health conditions. This study highlights the importance of monitoring HRQoL in people living with HIV due to the chronic nature of the disease. In this highly-treatment experienced group, disparities were found, particularly highlighting mental health as an area which needs more attention to improve the well-being of people living with HIV.

## Background

Health-related quality of life (HRQoL) is a multidimensional construct that relates one’s health to their overall feelings of well-being and perceived ability to function physically, mentally and emotionally [1]. HRQoL is distinguishable from other health metrics in that it is self-reported by the patient and represents an attempt to consider the impact of health on practical aspects of daily life. As such, measuring HRQoL is a crucial component in assessing and addressing the unmet needs of various populations, especially those with chronic illnesses, such as HIV.

Although people living with HIV (PLHIV) can expect a normal life expectancy through combination antiretroviral therapy (ART) when diagnosed and provided treatment promptly, they continue to face a disproportionate burden of chronic health problems, challenges of lifelong treatment and associated side-effects as well as psychological challenges including stigma and discrimination [2, 3]. Previous studies have typically shown lower HRQoL scores among people with HIV compared to the general population, and disparities in HRQoL scores between HIV sub-populations [4–7].

HRQoL data can be used to identify disparities between different populations and help to inform interventions that will ensure long-term retention in care, ART adherence and maintenance of good health [8]. Recently, there have been calls to formally consider good HRQoL as part of the “fourth 90” for people with HIV alongside the UNAIDS 90-90-90 targets to monitor the health system response to HIV [9].

This has led to greater interest in quantifying and measuring HRQoL among people with HIV; however, there is no agreed consensus on what tool is best to measure it [10]. The challenge is for HRQoL scales to be short and simple while also being sensitive enough to capture patients’ experience. There are a wide variety of HRQoL scales available, both generic and HIV-specific. Generic HRQoL scales apply to the general population and may not have been validated for use in HIV populations. EurQoL (EQ-5D) is a 5-item scale that has been widely used among general populations around the world and specifically among people living with HIV [11]. HIV-specific HRQoL scales include PROQOL-HIV [12], WHOQOL-HIV-BREF [13] and MOS-HIV [14]. However, the number of items in these scales range from 31 to 43 questions, making them burdensome for routine data collection at the clinic or population levels. The PozQoL scale is a new HIV-specific HRQoL instrument with 13 items, with promise for regular use in healthcare settings [15].

In this study, we assess the health-related quality of life of PLHIV in Romania and Spain, two European countries with contrasting epidemics: Romania with a younger (median age 33) population in eastern Europe and Spain with an ageing (median age 49) HIV population in western Europe. We use two brief, validated HRQoL instruments: a widely-used generic instrument, EQ-5D-5L (5-items), and an HIV-specific tool, PozQoL (13-items). We describe the distribution of EQ-5D domains among people living with HIV compared to the general population in both countries, and determine factors associated with a lower HRQoL.

This study is part of the European Commission’s Joint Action on Integration of Testing and Linkage to Care for HIV, viral hepatitis, tuberculosis, and sexual transmitted infections in Europe (INTEGRATE) [16].

## Methods

Infectious disease hospitals with HIV cohorts in Romania and Spain were identified through the INTEGRATE joint action [16]. The hospitals were self-selected after expressing interest in collecting data on HRQoL, but they also represented contrasting epidemic and therefore good comparators for using two different HRQoL instruments. A cross-sectional study was conducted among adult (≥ 18 years) PLHIV in Romania and Spain from October 2019 to March 2020. A structured questionnaire was developed by an expert advisory group of clinicians, social scientists, and PLHIV. The questionnaire was initially written in English and translated to Romanian and Spanish. Quality checks were performed through back-translation and piloting of the questionnaire in each language (n = 3–5 local volunteers) to ensure content validity and comprehension. Those patients unable to complete the questionnaire due to language or literacy were not eligible.

### Population and setting

#### Romania

The survey was conducted at the HIV Department of the "Victor Babes" Clinical Hospital of Infectious Diseases and Pneumophtisiology Craiova, which specializes in treating patients with infectious diseases. It serves a population of around 1.3 million and has an HIV outpatient clinic of 650 patients. HIV patients attend the clinic monthly to collect their prescription ART from the Hospital's HIV Department. They are evaluated clinically every three to six months. Consecutive HIV patients attending the clinic to collect their prescription during the study period were invited to complete the survey.

#### Spain

The survey was conducted at the Complejo Hospitalario de Navarra, Pamplona, and Hospital Reina Sofia, Tudela, located in Navarra, an autonomous community in northern Spain. Access to the Spanish healthcare system is universal. HIV care is provided within infectious diseases units located in outpatient clinics. ART is dispensed without charge in hospital pharmacies by specialized personnel.

HIV patients attend the HIV clinic for routine checkups every six months. Consecutive HIV patients attending the clinic during the study period were invited to participate.

### Measurement and evaluation of HRQoL

Two instruments were used to assess HRQoL: EQ-5D and PozQoL.

EQ-5D is a simple, generic 5-item instrument for measuring HRQoL and widely used in both clinical and observational studies [17–19]. Although not tailored for PLHIV, it has been used previously to measure HRQoL in this population [20]. The EQ-5D consists of five questions on mobility, self-care, usual activity, pain/discomfort, and anxiety/depression. Each question has five possible answer options, ranging from “no problems at all” to “severe problems” from which a single summary index can be derived, also known as a utility score. This ranges from 0 (representing a state worse than death) to 1 (representing perfect health).

PozQoL is a 13-item instrument designed specifically to measure HRQoL in PLHIV [15]. The instrument groups questions into four subscales covering: health concerns, psychological, social, and functional, which align with the World Health Organizations’ conceptualization of QoL [21]. Each question has five possible answer options, ranging from “not at all” to “extremely”. Scores range from 1 (low QoL) to 5 (very high QoL). In addition to its construct validity and reliability, the brevity of the tool makes it easier to administer than other HIV-specific instruments [15].

The two primary outcomes in this study were the EQ-5D utility score and the summary averaged PozQoL score.

The independent variables included demographic and socioeconomic factors: age, gender, sexual orientation, educational attainment, employment status, housing stability (stable housing defined as owning or renting and unstable housing defined as temporary accommodation, living with family or friends, or homeless), and having enough money for basic needs (always or not always). Ethnicity was collected in Romania and migrant status was collected in Spain after consultation with local teams to select the most culturally appropriate question. HIV-related characteristics included year of diagnosis, ART status, and adherence to ART. Health-related behaviours included self-rated health, diagnosed co-morbid physical mental health conditions, polypharmacy (use of five or more medications, including ART [22]), binge drinking (six or more drinks at one time for a woman or eight or more drinks at one time for a man [23]), smoking, and injecting drug use.

### Statistical analyses

The EQ-5D_utility_ score for Spain was constructed using the Hernandez et al. (2018) value set for Spain [24]. The utility score for Romania was constructed using the EuroQol cross-walk UK value set (in the absence of a national value set for Romania [25]). Distribution of EQ-5D domains were compared with population norms in Spain using the Hernandez dataset (2018) [24] and in Romania using the Paveliu et al. (2019) preliminary dataset [26] Records missing data on one or more of the five domains were excluded (Romania n = 0 and Spain n = 18).

PozQoL scores were computed as averages to retain records with missing values (0% of Romanian responses were missing ≥ 1 item and 18% of Spanish responses were missing ≥ 1 item). After recoding negatively worded items, the items within the same sub-scales were averaged together, per the scoring guide [27]. A total score was then created as an average of the completed questions. Participants with > 1 item missing on any one sub-scale excluded from the analysis (Spain n = 25 (6%)) and in Romania no participants had missing data. Score thresholds were set based on the PozQoL validation study for the full scale and sub-scales to assign participants as having very high, high, moderate, or low quality of life. The averaged summary score ranged from 0 to 5.

We initially performed descriptive analysis of the EQ-5D and PozQoL profile of respondents for each country. The overall EQ-5D utility score of PLHIV in both countries was compared to that of the respective general populations [28].

Both outcomes were treated as continuous and examined separately for each country. Exploratory analysis of EQ-5D utility score and PozQoL score using histograms found they were left skewed, particularly EQ-5D, which had a strong ceiling effect with 43% of all respondents reporting perfect health. Shapiro–Wilk and D'Agostino-Pearson tests confirmed both outcome variables were non-normally distributed (p < 0.0001 for both). For this reason, inferential analysis was performed using generalized linear modelling, with a γ distribution and log link. Univariable analysis was performed against each of the independent variables. Step-wise multiple linear regression was used to determine factors associated with poor HRQoL. Variables included in the final model included age and sex (a priori due to known associations with HRQoL) and those which were significant at the 0.10 level in the univariable analysis. In this analysis, negative effect estimates indicate worse health whereas positive values indicate better health.

A sensitivity analysis was performed for the EQ-5D utility score to test for variations caused by the ceiling effect. A logistic regression model was fitted with the EQ-5D utility score collapsed into a dichotomous variable representing perfect health (yes = 1 or no < 1). The model also found mental health a strong predictor but could not model self-rated health due to data loss. The main difference using logistic regression was age, which was no longer significant (p = 0.326). Given the similarity of the results, the linear model was retained to retain maximum data in the model while noting this difference.

All analyses were conducted using Stata version 15 (Stata Corp, College Station, Texas, USA).

## Results

### Descriptive statistics

We surveyed 170 PLHIV in Romania and 400 PLHIV in Spain (Table 1). The profile of respondents in Romania and Spain was very different. In Romania, the median age was 31 (range 18–67) compared to 52 (19–83) in Spain. In Spain, two-thirds (67%) were men, compared to less than half (44%) in Romania. Only 14% of respondents were in employment (full-time or part-time) in Romania compared to 60% of respondents in Spain. Most (86%) of the Spanish respondents had stable housing situations compared to 60% in Romania. Financial insecurity was commonly reported, with 38% of respondents in Romania and 53% in Spain saying they did not always have enough money to meet their basic needs. Nearly all respondents were on ART (99% in Romania and 99% in Spain; adherence was 86% in Romania and 99% in Spain.

**Table 1:** Characteristics of respondents and univariable comparison of mean EQ5D_utility_ scores in people living with HIV in Spain (n = 400) and Romania (n = 170)

	Romania					Spain				
	Cohort (%)	Ceiling effect*	Mean EQ5D_utility_			Cohort (%)	Ceiling effect*	Mean EQ5D_utility_		
		n (%)	mean	95% CI	p-value		n (%)	mean	95% CI	p-value
*Sociodemographic characteristics*										
Age (years): median (IQR)	31 (30–39)	34.7%	0.85	0.83–0.87	0.641	52 (43–57)	46.6%	0.88	0.87–0.90	**0.001**
Gender										
Men	43.5	41.9%	0.88	0.84–0.91	0.063	67.1	52.0%	0.89	0.87–0.91	**0.026**
Women	56.5	29.2%	0.83	0.80–0.86		31.9	35.3%	0.86	0.83–0.88	
Other	0.0					1.0	50.0%	0.94	0.87–1.00	
Ethnicity (Romania only)										
Romanian	84.7	37.5%	0.86	0.84–0.89	**0.045**		N/A			
Roma	15.5	19.2%	0.78	0.70–0.86						
Migrant status (Spain only)										
No		N/A				71.8	47.5%	0.89	0.87–0.90	0.569
Yes						28.3	44.2%	0.87	0.84–0.91	
Sex orientation										**0.002**
Heterosexual	97.6	34.0%	0.85	0.82–0.87	** < 0.001**	70.1	40.1%	0.86	0.84–0.88	
Homosexual/Bisexual	2.4	0.0%	0.80	0.70–0.86		29.9	62.4%	0.92	0.89–0.95	
Educational level										
No education	5.3	11.1%	0.75	0.66–0.85	0.088	1.8	60.0%	0.87	0.65–1.00	**0.0042**
Primary	7.1	33.3%	0.87	0.79–0.94		25.8	31.9%	0.82	0.78–0.86	
Secondary	62.4	33.0%	0.84	0.81–0.88		54.2	50.0%	0.90	0.88–0.92	
University +	25.3	44.2%	0.88	0.84–0.92		18.2	55.9%	0.91	0.87–0.95	
Employment										
Full- or Part-time	13.7	56.2%	0.93	0.89–0.97	** < 0.001**	60.1	57.4%	0.92	0.90–0.94	** < 0.001**
Unemployed	28.0	38.3%	0.88	0.85–0.92		22.9	30.9%	0.83	0.79–0.87	
Other (student, retired, disabled)	58.3	27.6%	0.81	0.78–0.85		17.0	28.6%	0.80	0.75–0.86	
Stable housing										
No	40.5	35.3%	0.85	0.81–0.89	0.828	13.7	30.8%	0.80	0.73–0.86	**0.005**
Yes (own or rent)	59.5	34.0%	0.85	0.82–0.88		86.3	49.1%	0.90	0.88–0.91	
Money for basic needs										
Not always	62.4	26.4%	0.82	0.78–0.85	** < 0.001**	53.2	34.3%	0.84	0.81–0.86	** < 0.001**
Always	37.7	48.4%	0.90	0.88–0.93		46.8	60.6%	0.93	0.91–0.95	
*HIV-related characteristics*										
Year of diagnosis										
After 2010	15.4	26.9%	0.86	0.81–0.91	0.538	31.4	56.1%	0.92	0.90–0.94	**0.003**
1995–2010	43.8	29.7%	0.83	0.79–0.87		33.3	46.9%	0.87	0.84–0.91	
Before 1995	40.8	42.0%	0.86	0.82–0.90		35.3	38.2%	0.86	0.83–0.89	
ART status										
No	1.2	0.0%	0.83	0.81–0.85	0.108	0.8	66.7%	0.86	0.59–1.00	0.859
Yes	98.8	35.1%	0.85	0.83–0.88		99.2	46.5%	0.88	0.87–0.90	
Adherence to ART										
Fully adherent	83.9	38.3%	0.86	0.83–0.89	0.301	99.2	48.7%	0.89	0.87–0.90	0.435
Not fully adherent	16.1	18.2%	0.82	0.75–0.89		0.9	33.3%	0.83	0.65–1.00	
*Health-related characteristics*										
Self-rated health										
Bad	5.3	0.0%	0.67	0.56–0.77	** < 0.001**	3.3	0.0%	0.57	0.45–0.69	** < 0.001**
Fair	22.4	13.2%	0.76	0.70–0.82		16.0	5.1%	0.72	0.67–0.76	
Good	72.4	43.9%	0.89	0.87–0.91		80.7	56.9%	0.93	0.92–0.94	
Physical health problems										
No	70.9	44.4%	0.89	0.87–0.92	** < 0.001**	70.7	54.4%	0.91	0.90–0.93	** < 0.001**
Yes	29.1	14.6%	0.75	0.69–0.80		29.3	29.3%	0.81	0.77–0.85	
Mental health problems										
No	93.5	35.2%	0.85	0.83–0.88	0.292	80.5	58.3%	0.93	0.92–0.94	** < 0.001**
Yes	6.5	27.3%	0.80	0.70–0.90		19.5	4.3%	0.70	0.65–0.75	
Total number of comorbidities										
None	75.9	39.5%	0.87	0.85–0.90	** < 0.001**	71.9	55.0%	0.92	0.91–0.94	** < 0.001**
1	16.5	21.4%	0.79	0.72–0.86		18.4	25.0%	0.82	0.78–0.86	
2+	7.7	15.4%	0.78	0.68–0.88		9.7	27.0%	0.78	0.70–0.85	
Polypharmacy										
No	88.2	38.7%	0.87	0.84–0.89	**0.006**	78.0	52.9%	0.91	0.89–0.92	** < 0.001**
Yes	11.8	5.0%	0.72	0.62–0.82		22.0	23.2%	0.80	0.75–0.84	
*Behavioral characteristics*										
Binge drinking										
Weekly	3.6	50.0%	0.83	0.80–0.87	** < 0.001**	6.0	30.4%	0.88	0.84–0.92	**0.047**
Monthly	6.5	54.6%	0.87	0.83–0.91		6.5	39.1%	0.84	0.77–0.91	
Less than once a month	18.9	34.4%	0.93	0.87–0.98		24.7	56.5%	0.92	0.89–0.94	
Never	71.0	32.5%	0.94	0.89–0.99		62.9	46.8%	0.88	0.86–0.90	
Tobacco use										
No	65.9	38.4%	0.87	0.84–0.89	0.076	53.9	54.8%	0.91	0.89–0.93	** < 0.001**
Yes	34.1	27.6%	0.82	0.77–0.87		46.2	38.3%	0.85	0.83–0.88	
Injecting drugs use										
Never	97.6	34.6%	0.85	0.82–0.87	0.159	77.6	51.4%	0.90	0.88–0.91	0.098
Yes, previously	1.8	33.3%	0.91	0.81–1.00		18.0	35.3%	0.85	0.82–0.89	
Yes, currently	0.6	0.0%	0.88	–		4.4	23.5%	0.85	0.77–0.93	

*Ceiling effect is the percentage of participants that reported perfect health (EQ5D_utility_ = 1)

### EQ-5D score, distribution and general population comparisons

One a scale of 0 to 1, the mean EQ-5D_utility_ score among people with HIV in Spain was 0.88 (SD = 0.16) compared to 0.91 (SD ± 0.15) in the general population. In Romania, the mean EQ-5D_utility_ score among people with HIV was 0.85 (SD ± 0.16).

**Fig. 1:**
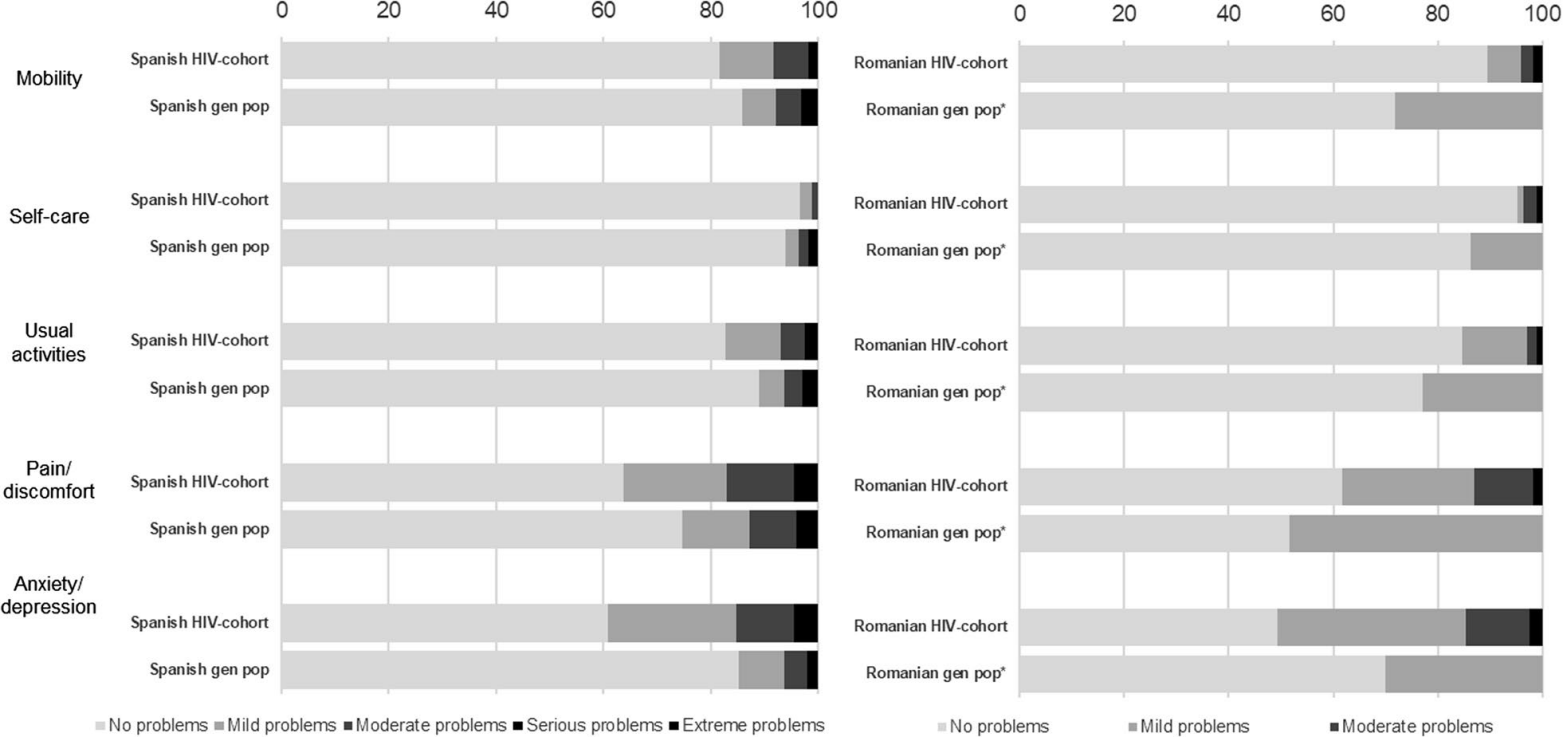
Comparison of the distribution of the EQ-5D domains in the HIV population and the general population in Spain (left) and Romania (right). *For Romania, the general population comparison data for each domain was only available at an aggregated level. Therefore, the green bar represents no symptoms, and the red bar represents any symptom (mild, moderate, severe, or extreme)

Figure 1 shows the distribution of the EQ-5D domains in Spain and Romania. In both countries, the most affected domain was anxiety/depression (Spain: 38% reported some problems vs 15% in general Spanish population; Romania: 50% reported some problems vs 30% in the general Romanian population).

Across the other EQ-5D domains, such as pain/discomfort and mobility, PLHIV in Spain reported more problems compared to the general population, whereas PLHIV in Romania reported fewer problems overall.

### PozQoL score, distribution and general population comparisons

**Fig. 2:**
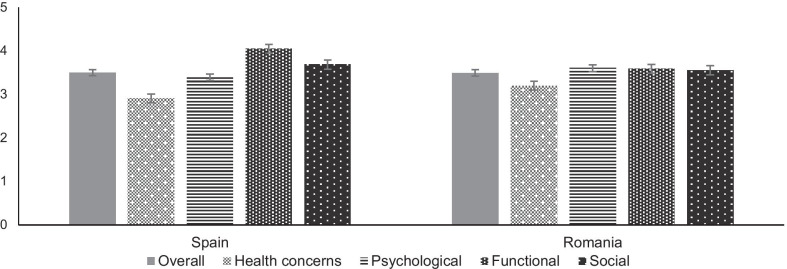
Summary averaged PozQoL scores and sub-scales in Spain (left) and Romania (right)

Figure 2 shows the distribution of the summary averaged PozQoL scores for each country. In Spain, the summary average PozQoL score was 3.5 (SD ± 0.71) with a range of 1.4 to 5.0 (median 3.6 IQR (3.1–4.0). In Romania, the summary averaged PozQoL score was 3.50 ± 0.79 with a range of 1.4–5.0 (median 3.5 IQR (3.0–4.2). In both countries, the lowest summary averaged score was given for the health concerns sub-scale (2.9 in Spain and 3.2 in Romania).

**Fig. 3:**
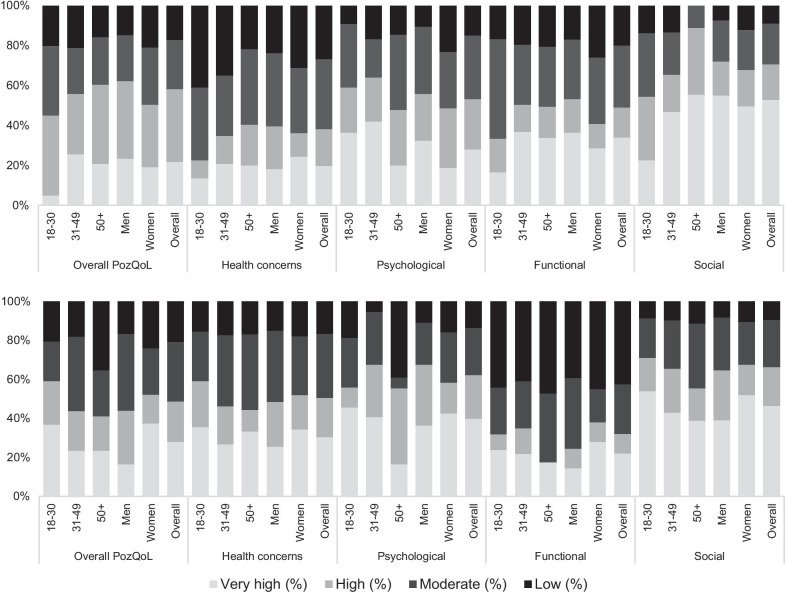
Distribution of PozQoL quality of life categories by age and gender and sub-scales in Spain (top) and Romania (bottom)

PozQoL scores, overall and for each sub-scale were classified into ‘very high’, ‘high’, ‘moderate’, and ‘low’ quality of life using the pre-defined cut-off values from the PozQoL protocol (Fig. 3). In Spain, 58.2% had ‘high’ or ‘very high QoL’, compared to 48.8% in Romania. In Spain, the PozQoL health concerns sub-scale was most affected, with 1 in 4 (26.7%) PLHIV scoring in the ‘low’ QoL category. Women scored worse on the PozQoL psychological and functional sub-scales, and younger adults (aged 18–30 years) scored worse on PozQoL health concerns subscale.

In Romania, the PozQoL functional sub-scale was most affected, with 42% of respondents scoring in the ‘low’ QoL category. In both Spain and Romania, the PozQoL social subscale was least affected with only 9% scoring in the ‘low’ QoL category.

### Factors associated with worse HRQoL in people living with HIV

In univariable analysis of the EQ-5D scale, several factors were significantly associated with a lower mean EQ-5D_utility_ score (Table 1). However, in the adjusted model for Spain only poor self-rated health, older age, and having ever been diagnosed with a mental health condition were predictive of a lower EQ-5D_utility_ score (Table 2).

**Table 2:** Multiple linear regression predicting lower HRQoL score for EQ-5D utility score and PozQoL score in people living with HIV in Romania and Spain

	Model 1: Adjusted model for mean EQ-5D utility score				Model 2: Adjusted model for summary averaged PozQoL score			
	Romania		Spain		Romania		Spain	
	Coefficient	Robust standard error	Coefficient	Robust standard error	Coefficient	Robust standard error	Coefficient	Robust standard error
Model								
Age, years			− 0.00*	0.00				
Homosexual/bisexual					− 0.23**	0.09		
Economically Inactive^†^	− 0.05	0.03	0.00	0.03	− 0.11*	0.05	0.02	0.03
Money for basic needs, not always							− 0.08***	0.02
Self-rated health, very bad or bad	− 0.26**	0.09	− 0.43***	0.12			− 0.15	0.11
Self-rated health, fair	− 0.13**	0.05	− 0.23***	0.04			− 0.10**	0.03
Mental health diagnosis, ever			− 0.16***	0.03			− 0.14***	0.04
Physical co-morbidity	− 0.17***	0.04						

Negative coefficients indicate worse HRQoL whereas positive values indicate better HRQoL; explanatory variables selected a priori (age and gender) and through univariable analysis where p<0.05. Only explanatory variables with statistically significant results are shown. The Romania EQ-5D model was adjusted for age, Roma ethnicity, gender, education, employment, money for basic needs, mental health diagnosis, binge drinking, and ever injecting drug use. The Spain EQ-5D model was adjusted for gender, education, employment, month for basic needs, physical comorbidity, polypharmacy, smoking, and ever injecting drug use. The Romania PozQoL model was adjusted for age, Roma ethnicity, gender, employment, money for basic needs, self-rated health, mental health diagnoses, physical comorbidity and polypharmacy. The Spain PozQoL model was adjusted for age, migrant status, gender, and employment

*p < 0.05; **p < 0.01, ***p < 0.001

^†^ Retired, student, disabled

Likewise, in Romania, several factors were statistically significant in univariable analysis of the EQ-5D_utility_ score, including alcohol and injecting drug use. However, in the fully adjusted model only poor self-rated heath and presence of a diagnosed co-morbid physical health condition were predictive of worse HRQoL. In contrast to Spain, in Romania, mental health conditions and older age were not significantly associated with a lower mean EQ-5D_utility_ score in univariable or multivariable analysis.

In the analysis of the summary averaged PozQoL score, four factors were consistently associated with lower scores across most PozQoL sub-scales and in both countries in univariable analysis: gay/bisexual sexual orientation, not having money for basic needs, poor self-rated health, and having ever been diagnosed with a mental health condition (Table 3, results of PozQoL sub-scales in Appendices Tables 4, 5).

**Table 3:** Characteristics of respondents and univariable comparison of mean PozQoL scores in people living with HIV in Spain (n = 400) and Romania (n = 170)

	Romania			Spain		
	PozQoL mean score			PozQoL mean score		
	Mean	95% CI	p value	Mean	95% CI	p value
*Sociodemographic characteristics*						
Age (years): median (IQR)	3.50	3.38–3.62	0.531	3.50	3.43–3.57	**0.037**
Gender						
Men	3.48	3.33–3.63	0.772	3.57	3.48–3.65	**0.0195**
Women	3.51	3.33–3.69		3.38	3.24–3.51	
Other				2.82	1.42–4.22	
Ethnicity (Romania only)						
Romanian	3.57	3.44–3.70	**0.002**			
Roma	3.10	2.84–3.36				
Migrant status (Spain only)						
Born in Spain				3.56	3.48–3.64	**0.0117**
Born abroad				3.35	3.20–3.50	
Sex orientation						
Heterosexual	3.50	3.38–3.63	** < 0.001**	3.47	3.38–3.53	0.423
Homosexual/Bisexual	2.95	2.53–3.36		3.53	3.41–3.66	
Educational level						
No education	3.06	2.54–3.58	0.193	3.04	2.18–3.90	0.1372
Primary	3.28	2.80–3.76		3.41	3.25–3.57	
Secondary	3.50	3.36–3.64		3.53	3.43–3.63	
University +	3.65	3.38–3.92		3.60	3.46–3.75	
Employment						
Full- or Part-time	3.86	3.58–4.15	** < 0.001**	3.58	3.49–3.67	**0.0131**
Unemployed	3.69	3.51–3.87		3.27	3.09–3.45	
Other (student, retired, disabled)	3.30	3.13–3.46		3.47	3.29–3.65	
Stable housing						
No	3.52	3.33–3.71	0.744	3.41	3.21–3.61	0.349
Yes (own or rent)	3.48	3.32–3.64		3.51	3.44–3.59	
Money for basic needs						
Not always	3.38	3.23–3.53	**0.010**	3.29	3.18–3.40	**0.0000**
Always	3.69	3.50–3.88		3.73	3.64–3.81	
*HIV-related characteristics*						
Year of diagnosis						
After 2010	3.39	3.09–3.69	0.456	3.47	3.34–3.61	0.474
1995–2010	3.46	3.26–3.66		3.45	3.31–3.60	
Before 1995	3.58	3.41–3.75		3.56	3.44–3.68	
ART status						
No	3.15	2.70–3.61	0.570	2.90	1.46–4.33	**0.045**
Yes	3.50	3.38–3.62		3.50	4.43–3.57	
Adherence to ART						
Fully adherent	3.52	3.37–3.67	0.738	3.55	3.47–3.62	0.446
Not fully adherent	3.58	3.26–3.90		3.26	1.33–5.18	
*Health-related characteristics*						
Self-rated health						
Bad	3.07	2.70–3.44	** < 0.001**	2.82	2.25–3.39	** < 0.001**
Fair	3.15	2.91–3.39		3.09	2.90–3.27	
Good	3.63	3.50–3.77		3.61	3.53–3.68	
Physical health problems						
No	3.61	3.47–3.74	**0.015**	3.57	3.48–3.65	**0.019**
Yes	3.25	3.00–3.50		3.36	3.22–3.51	
Mental health problems						
No	3.54	3.42–3.66	**0.019**	3.65	3.58–3.73	** < 0.001**
Yes	2.86	2.33–3.39		3.03	2.86–3.20	
Total number of comorbidities						
None	3.56	3.43–3.69	0.500	3.57	3.48–3.66	0.1427
1	3.36	3.00–3.71		3.47	3.29–3.66	
2 +	3.29	2.83–3.76		3.36	3.03–3.70	
Polypharmacy						
No	3.55	3.42–3.67	**0.039**	3.53	3.44–3.61	0.093
Yes	3.12	2.74–3.50		3.38	3.24–3.53	
*Behavioral characteristics*						
Binge drinking						
Weekly	3.51	3.36–3.66	0.303	3.45	3.15–3.76	0.3978
Monthly	3.52	3.25–3.79		3.38	3.09–3.66	
Less than once a month	3.17	2.81–3.54		3.60	3.46–3.75	
Never	3.54	3.44–3.64		3.49	3.40–3.58	
Tobacco use						
No	3.55	3.40–3.70	0.193	3.53	3.43–3.62	0.504
Yes	3.39	3.20–3.58		3.48	3.37–3.59	
Injecting drugs use						
Never	3.48	3.36–3.60	** < 0.001**	3.50	3.41–3.58	0.069
Yes, previously	4.41	4.10–4.72		3.62	3.47–3.78	
Yes, currently	3.46	–		3.24	2.91–3.58	

In the fully adjusted model of the summary averaged PozQoL score, in Spain, poor self-rated health, ever diagnosed with a mental health condition, and not having money for basic needs were significantly associated with lower PozQoL scores, whereas in Romania a different set of factors emerged: gay/bisexual sexual orientation, employment status (specifically those classed as disabled), and presence of a mental health condition were significantly associated with lower PozQoL scores (Table 2). No factors related to clinical HIV infection, such as ART status and adherence and time since HIV diagnosis, were associated with worse HRQoL by either the EQ-5D or PozQoL measure.

Only explanatory variables with statistically significant results are shown in Table 2. The Romania EQ-5D model was adjusted for age, Roma ethnicity, gender, education, employment, money for basic needs, mental health diagnosis, binge drinking, and ever injecting drug use. The Spain EQ-5D model was adjusted for gender, education, employment, month for basic needs, physical comorbidity, polypharmacy, smoking, and ever injecting drug use. The Romania PozQoL model was adjusted for age, Roma ethnicity, gender, employment, money for basic needs, self-rated health, mental health diagnoses, physical comorbidity and polypharmacy. The Spain PozQoL model was adjusted for age, migrant status, gender, and employment.

## Discussion

This cross-sectional study examined HRQoL in PLHIV in Romania and Spain in 2019–2020. The results suggest that HRQoL for people living with HIV is slightly lower than the general population but statistically comparable. Overall, PLHIV in Romania reported lower EQ-5D utility scores compared to Spain but provided identical PozQoL scores overall. Importantly, these results showed that HRQoL between countries differ, highlighting the importance of having country-specific measurements of HRQoL rather than assuming similar scores for persons with HIV living in different countries.

In this sample of PLHIV with nearly all (> 98%) respondents on ART, no HIV clinical factors were found to be associated with poor HRQoL. This is a positive reflection of the incredible advances in HIV treatment globally as well as the quality of the HIV care provision in both countries health systems. Detailed analysis of the HRQoL of people either not taking or non-adherent to ART was therefore limited by small numbers.

Psychological issues, poor health status, and having concerns about one’s health were found to be important predictors of poor HRQoL. This aligns with previous research which has found PLHIV disproportionately experience poorer mental health [29], caused by factors such as coping with a recent HIV diagnosis, the impact of a diagnosis on relationships, stigmatisation and experiences of discrimination, social isolation and loneliness, and the experience of living with a chronic illness [7, 30, 31]. Additionally, marginalized populations who are also at risk of HIV such as gay and bisexual populations, migrants, drug users, and prisoners also experience poorer mental health [32].

Our study found that social and structural factors, such as sexuality, financial instability and employment, were important predictors of HRQoL, particularly in Romania. This reflects the cultural and societal differences between the two countries: Spain, a high-income, socially progressive country and Romania, a developing/emerging economy with a conservative stance on social issues such as homosexuality. These findings underscore the influence of social norms, legal and economic status, and other factors on health, both directly and indirectly. This effect may be magnified in people with HIV, who are likely to experience stigma and discrimination due to their HIV status.

This study is novel in that it employed two different tools to examine HRQoL in PLHIV. While the EQ-5D instrument provides valuable comparisons between PLHIV and the general population, we found the EQ-5D_utility_ score was highly skewed in our population with strong ceiling effects. This made the analysis challenging and meant that nearly half the sample were indistinguishable using the five basic domains. The PozQoL tool allowed a more nuanced analysis, with a more even distribution. While the summary mean PozQoL score was identical for both countries, differences were seen in the sub-scales, showing the value of using HIV-specific tools to capture issues of relevance for different groups of PLHIV.

This study has several limitations. First, there is no EQ-5D value set available for Romania, which would allow calculation of an EQ-5D utility score based on the country’s population. Therefore, we used a United Kingdom value set to calculate Romanian EQ-5D utility scores, as has been done in previous studies [33, 34] and has been recommended for countries without national value sets to ensure comparability [35]. Using a value set from a different country may bias the utility score given the health and social differences between both countries. A Romanian value set is forthcoming, and the distribution of the EQ-5D domains presented are based on the data collected for this study [36].

Secondly, the cross-sectional nature of this study makes it difficult to determine causal relationships that might influence HRQoL, as response to many of the survey questions can change over time. This highlights the need for longitudinal research to better explore this area. Finally, the results of the study might not be generalizable to all PLHIV in Romania and Spain. Compared to the latest data, our sample was similar in age profile, but our sample was more treatment experienced (nationally, 82% in Romania and 93% in Spain) and included more women (56% in our sample vs 38% in Romania; 32% in our sample vs 16% in Spain) [37, 38].

Monitoring HRQoL is a vital component in assessing whether health systems meet the needs of patients and to ensure patients remain engaged in long-term care. As part of this project, a parallel HRQoL survey was piloted in people undergoing treatment for hepatitis C infection. This survey experienced challenges in recruitment. In contrast to HIV, hepatitis C is curable and with widespread access to direct-acting antivirals in Europe. However, the pool of eligible participants became too small to recruit a sample large enough for meaningful comparison. HRQoL surveys are likely to be more feasible in HIV populations who attend healthcare services at regular intervals. Additional research is needed to understand whether HRQoL surveys can be feasibly applied in other infectious disease populations.

## Conclusion

This study highlights the importance of understanding HRQoL in people living with HIV through the use of two HRQoL assessment tools. EQ-5D scores indicated a disproportionate burden of mental health symptoms impacting quality of life in people living with HIV, while the PozQoL scores indicated that concerns about future health are prevalent. Poor self-rated health was a good predictor of poor quality of life overall and may be considered as a screening tool for additional interventions to assess the holistic needs of people living with HIV.

## Appendix 1

See Table 4.

**Table 4:** PozQoL sub-scale scores and univariable analysis people living with HIV in Romania

	PozQoL Health concerns			PozQoL Psychological			PozQoL Functional			PozQoL Social		
	Mean	95% CI	p value	Mean	95% CI	p value	Mean	95% CI	p value	Mean	95% CI	p value
*Sociodemographic characteristics*												
Age (years): median (IQR)	3.20	3.03–3.36	0.418	3.61	3.48–3.74	0.076	3.59	3.42–3.76	0.739	3.56	3.40–3.72	0.591
Gender												
Men	3.13	2.90–3.38	0.542	3.69	3.48–3.84	0.473	3.57	3.35–3.79	0.863	3.47	3.26–3.69	0.343
Women	3.24	3.02–3.46		3.57	3.38–3.75		3.60	3.36–3.84		3.62	3.40–3.85	
Ethnicity												
Romanian	3.29	3.11–3.46	**0.004**	3.71	3.57–3.85	** < 0.001**	3.63	3.44–3.81	0.235	3.60	3.42–3.77	0.212
Roma	2.69	2.34–3.04		3.02	2.72–3.32		3.38	3.04–3.73		3.33	2.96–3.71	
Sex orientation												
Heterosexual	3.22	3.05–3.28	** < 0.001**	3.60	3.48–3.73	** < 0.001**	3.60	3.42–3.77	**0.004**	3.56	3.40–3.72	** < 0.001**
Homosexual/ bisexual	1.44	0.57–2.32		3.42	1.69–5.13		3.00	2.34–3.66		3.78	2.24–5.31	
Educational level												
No education	3.07	2.29–3.86	0.946	2.81	2.33–3.28	**0.001**	3.07	2.36–3.79	0.365	3.37	2.62–4.12	0.918
Primary	3.08	2.44–3.73		3.17	2.61–3.72		3.47	2.92–4.02		3.44	3.84–4.05	
Secondary	3.19	3.10–3.39		3.62	3.47–3.77		3.57	3.37–3.77		3.56	3.37–3.76	
University +	3.26	2.91–3.62		3.86	3.59–4.13		3.78	3.39–4.17		3.61	3.26–3.96	
Employment												
Full- or part-time	3.36	2.96–3.77	0.070	3.95	3.65–4.24	**0.014**	4.09	3.6–4.57	** < 0.001**	4.01	3.64–4.39	**0.002**
Unemployed	3.42	3.15–3.68		3.70	3.50–3.91		3.89	3.61–4.16		3.76	3.49–4.03	
Other (student, retired, disabled)	3.03	2.81–3.26		3.46	3.27–3.65		3.30	3.09–3.52		3.33	3.12–3.56	
Stable housing												
No	3.20	2.93–3.46	0.878	3.54	3.33–3.75	0.366	3.71	3.46–3.96	0.240	3.63	3.39–3.86	0.474
Yes (own or rent)	3.17	2.97–3.37		3.66	3.49–3.83		3.51	3.28–3.74		3.51	3.29–3.73	
Money for basic needs												
Not always	3.08	2.88–3.29	0.070	3.47	3.30–3.64	**0.004**	3.76	3.48–4.04	0.112	3.45	3.25–3.65	0.074
Always	3.38	3.13–3.63		3.83	3.65–4.02		3.48	3.27–3.69		3.74	3.48–4.00	
*HIV-related characteristics*												
Year of diagnosis												
After 2010	3.23	2.76–3.70	0.689	3.44	3.15–3.73	0.494	3.36	2.93–3.78	0.190	3.51	3.13–3.90	0.650
1995–2010	3.12	2.88–3.36		3.65	3.44–3.85		3.50	3.23–3.78		3.49	3.23–2.76	
Before 1995	3.27	3.02–3.52		3.63	3.42–3.83		3.75	3.51–4.00		3.65	3.42–3.88	
ART status												
No	3.00	–	**0.013**	2.88	2.13–3.62	**0.015**	3.50	2.51–4.49	0.809	3.33		**0.004**
Yes	3.20	3.03–3.36		3.61	3.48–3.75		3.60	3.42–3.76		3.56	3.40–3.72	
Adherence to ART												
Fully adherent	3.14	2.93–3.34	0.451	3.71	3.56–3.87	0.372	3.59	3.37–3.81	0.630	3.57	3.38–3.77	0.359
Not fully adherent	3.31	2.87–3.77		3.52	3.13–3.91		3.70	3.30–4.10		3.80	3.35–4.22	
*Health-related characteristics*												
Self-rated health												
Bad	3.07	2.46–3.69	0.111	2.58	2.17–2.99	** < 0.001**	3.33	2.84–3.82	**0.001**	3.44	2.85–4.05	**0.029**
Fair	2.88	2.53–3.23		3.36	3.07–3.65		3.11	2.81–3.39		3.18	2.86–3.49	
Good	3.30	3.12–3.49		3.76	3.61–3.90		3.76	3.55–4.0		3.68	3.50–3.87	
Physical health problems												
No	3.33	3.14–3.52	**0.039**	3.67	3.52–3.81	0.190	3.72	3.52–3.91	**0.035**	3.69	3.51–3.87	**0.024**
Yes	2.94	2.64–3.25		3.44	3.14–3.74		3.29	3.0–3.62		3.25	2.93–3.58	
Mental health problems												
No	3.22	3.06–3.39	0.291	3.66	3.53–3.80	**0.003**	3.63	3.45–3.80	**0.049**	3.60	3.44–3.77	**0.043**
Yes	2.82	2.10–3.54		2.75	2.21–3.29		3.03	2.48–3.58		2.88	2.23–3.53	
Total number of comorbidities												
None	3.25	3.07–3.43	0.258	3.69	3.54–3.82	**0.011**	3.65	3.46–3.83	0.185	3.62	3.44–3.79	0.259
1	3.02	2.59–3.46		3.56	3.19–3.94		3.44	2.98–3.90		3.35	2.88–3.83	
2 +	3.02	2.50–3.55		2.90	2.40–3.41		3.33	2.83–3.83		3.41	2.85–3.97	
Polypharmacy												
No	3.24	3.06–3.41	0.134	3.67	3.53–3.81	**0.008**	3.62	3.43–3.79	0.380	3.62	3.46–3.79	**0.048**
Yes	2.90	2.50–3.30		3.11	2.74–3.49		3.38	2.90–3.87		3.07	2.56–3.57	
*Behavioral characteristics*												
Binge drinking												
Weekly	3.17	2.61–3.72	0.579	3.34	3.10–3.65	0.314	4.22	4.08–4.36	** < 0.001**	3.44	2.86–4.02	0.099
Monthly	2.76	2.14–3.38		3.80	3.34–4.25		3.21	2.74–3.68		2.73	2.09–3.36	
Less than once a month	3.27	2.84–3.70		3.64	3.34–3.94		3.52	3.12–3.91		3.60	3.26–3.94	
Never	3.21	3.02–3.40		3.59	3.43–3.75		3.60	3.39–3.80		3.62	3.43–3.81	
Tobacco use												
No	3.25	3.05–3.45	0.354	3.63	3.47–3.78	0.667	3.64	3.42–3.87	0.298	3.66	3.46–3.85	0.090
Yes	3.09	2.82–3.35		3.56	3.32–3.81		3.48	3.25–3.71		3.37	3.10–3.64	
Injecting drugs use												
Never	3.18	3.02–3.35	** < 0.001**	3.59	3.46–3.72	** < 0.001**	3.57	3.40–3.74	** < 0.001**	3.54	3.38–3.70	** < 0.001**
Yes, previously	4.33	3.33–5.34		4.42	4.09–4.74		4.11	2.67–5.55		4.78	4.34–5.22	
Yes, currently	2.67	–		3.75	–		4.33	–		3.00	–	

## Appendix 2

See Table 5.

**Table 5:** PozQoL sub-scale scores and univariable analysis people living with HIV in Spain

	PozQoL Health concerns			PozQoL Psychological			PozQoL Functional			PozQoL Social		
	Mean	95% CI	p value	Mean	95% CI	p value	Mean	95% CI	p value	Mean	95% CI	p value
*Sociodemographic characteristics*												
Age (years): median (IQR)	3.50	3.43–3.57	**0.037**	2.91	2.81–3.01	** < 0.001**	3.40	3.33–3.48	**0.010**	4.05	3.95–4.15	0.296
Gender												
Men	3.57	3.48–3.65	**0.0195**	2.92	2.80–3.04	** < 0.001**	3.49	3.40–3.59	**0.004**	4.13	4.02–4.25	0.0693
Women	3.38	3.24–3.51		2.91	2.72–3.10		3.22	3.08–3.36		3.88	3.69–4.07	
Other	2.82	1.42–4.22		1.75	1.26–2.24		3.02	2.17–3.87		3.67	2.18–5.16	
Ethnicity												
Romanian	3.56	3.48–3.64	**0.0117**	2.99	2.88–3.10	**0.013**	3.40	3.31–3.48	0.765	4.09	3.98–4.20	0.219
Roma	3.35	3.20–3.50		2.68	2.46–2.89		3.43	3.26–3.59		3.95	3.75–4.14	
Sex orientation												
Heterosexual	3.47	3.38–3.53	0.423	2.96	2.84–3.09	**0.014**	3.31	3.22–3.40	** < 0.001**	3.95	3.82–4.07	**0.006**
Homosexual/bisexual	3.53	3.41–3.66		2.7	2.52–2.87		3.63	3.48–3.78		4.23	4.07–4.39	
Educational level												
No education	3.04	2.18–3.90	0.1372	2.22	1.17–3.33	0.6316	3.46	3.01–3.91	**0.001**	3.33	2.09–4.58	0.1065
Primary	3.41	3.25–3.57		2.92	2.71–3.14		3.20	3.05–3.35		3.96	3.74–4.17	
Secondary	3.53	3.43–3.63		2.95	2.81–3.09		3.40	3.29–3.51		4.07	3.93–4.20	
University +	3.60	3.46–3.75		2.88	2.68–3.08		3.65	3.49–3.82		4.24	4.07–4.42	
Employment												
Full- or part-time	3.58	3.49–3.67	**0.0131**	2.89	2.76–3.02	0.4066	3.52	3.43–3.62	** < 0.001**	4.18	4.07–4.30	**0.0023**
Unemployed	3.27	3.09–3.45		2.83	2.57–3.09		3.19	3.01–3.37		3.69	3.44–3.94	
Other (student, retired, disabled)	3.47	3.29–3.65		3.04	2.82–3.26		3.19	3.00–3.38		3.96	3.70–4.23	
Stable housing												
No	3.41	3.21–3.61	0.349	3.01	2.75–3.26	0.397	3.10	2.89–3.32	**0.004**	3.94	3.65–4.24	0.431
Yes (own or rent)	3.51	3.44–3.59		2.89	2.78–3.00		3.45	3.36–3.53		4.07	3.97–4.18	
Money for basic needs												
Not always	3.29	3.18–3.40	**0.0000**	2.78	2.63–2.92	**0.0051**	3.19	3.07–3.30	** < 0.001**	3.76	3.61–3.92	** < 0.001**
Always	3.73	3.64–3.81		3.07	2.92–3.20		3.64	3.55–3.74		4.34	4.24–4.45	
*HIV-related characteristics*												
Year of diagnosis												
After 2010	3.47	3.34–3.61	0.474	2.62	2.44–2.81	**0.001**	3.50	3.34–3.67	0.175	4.14	3.97–4.31	0.330
1995–2010	3.45	3.31–3.60		2.92	2.72–3.11		3.44	3.30–3.58		3.96	3.76–4.16	
Before 1995	3.56	3.44–3.68		3.08	2.93–3.24		3.32	3.19–3.45		4.00	3.84–4.16	
ART status												
No	2.90	1.46–4.33	**0.045**	1.11	0.89–1.33	** < 0.001**	3.25	2.11–4.39	0.748	4.44	3.35–5.54	0.354
Yes	3.50	4.43–3.57		2.92	2.82–3.02		3.41	3.33–3.48		4.04	3.94–4.14	
Adherence to ART												
Fully adherent	3.55	3.47–3.62	0.446	2.94	2.84–3.05	**0.015**	3.43	3.35–3.52	0.487	4.09	3.99–4.20	0.970
Not fully adherent	3.26	1.33–5.18		2.44	2.01–2.88		3.08	1.93–4.23		4.11	2.95–5.27	
*Health-related characteristics*												
Self-rated health												
Bad	2.82	2.25–3.39	** < 0.001**	2.69	2.07–3.32	0.551	2.42	1.92–2.93	** < 0.001**	3.10	2.45–3.75	** < 0.001**
Fair	3.09	2.90–3.27		2.81	2.57–3.05		2.94	2.79–3.09		3.43	3.14–3.72	
Good	3.61	3.53–3.68		2.93	2.82–3.04		3.54	3.46–3.62		4.21	4.11–4.31	
Physical health problems												
No	3.57	3.48–3.65	0.019	2.97	2.84–3.09	0.133	3.51	3.41–3.60	** < 0.001**	4.11	3.99–4.23	0.089
Yes	3.36	3.22–3.51		2.80	2.63–2.97		3.16	3.01–3.32		3.91	3.72–4.11	
Mental health problems												
No	3.65	3.58–3.73	** < 0.001**	3.01	2.89–3.12	**0.014**	3.55	3.47–3.63	** < 0.001**	4.25	4.16–4.35	** < 0.001**
Yes	3.03	2.86–3.20		2.68	2.46–2.90		2.89	2.71–3.06		3.40	3.14–3.68	
Total number of comorbidities												
None	3.57	3.48–3.66	0.1427	2.91	2.78–3.03	0.6356	3.53	3.43–3.62	**0.005**	4.13	4.01–4.25	0.130
1	3.47	3.29–3.66		2.98	2.74–3.23		3.25	3.08–3.43		3.95	3.69–4.20	
2 +	3.36	3.03–3.70		2.78	2.45–3.11		3.14	2.81–3.47		3.78	3.40–4.15	
Polypharmacy												
No	3.53	3.44–3.61	0.093	2.90	2.78–3.02	0.991	3.47	3.38–3.55	**0.005**	4.07	3.95–4.18	0.329
Yes	3.38	3.24–3.53		2.90	2.70–3.10		3.18	2.99–3.36		3.95	3.74–4.16	
*Behavioral characteristics*												
Binge drinking												
Weekly	3.45	3.15–3.76	0.3978	2.89	2.47–3.32	0.3101	3.39	3.04–3.73	0.648	4.05	3.68–4.41	0.576
Monthly	3.38	3.09–3.66		2.70	2.29–3.10		3.29	2.92–3.67		3.94	3.52–4.36	
Less than once a month	3.60	3.46–3.75		3.07	2.86–3.27		3.49	3.33–3.64		4.17	3.99–4.36	
Never	3.49	3.40–3.58		2.88	2.75–3.01		3.39	3.29–3.48		4.03	3.90–4.16	
Tobacco use												
No	3.53	3.43–3.62	0.504	2.83	2.69–2.97	0.093	3.48	3.38–3.58	**0.047**	4.15	4.02–4.27	**0.047**
Yes	3.48	3.37–3.59		3.00	2.86–3.15		3.32	3.20–3.44		3.95	3.80–4.10	
Injecting drugs use												
Never	3.50	3.41–3.58	0.069	2.85	2.74–2.97	**0.026**	3.44	3.35–3.53	0.191	4.09	3.97–4.20	0.177
Yes, previously	3.62	3.47–3.78		3.18	2.96–3.40		3.37	3.19–3.55		4.03	3.81–4.25	
Yes, currently	3.24	2.91–3.58		2.86	2.32–3.41		3.04	2.62–3.47		3.71	3.33–4.09	

## Abbreviations

ART: Antiretroviral therapy
EQ-5D: EurQoL five dimensions questionnaire
HRQoL: Health-related quality of life
PLHIV: People living with HIV
UK: United Kingdom
HIV: Human Immunodeficiency virus
QoL: Quality of Life
INTEGRATE: The European Commission’s Joint Action on Integration of Testing and Linkage to Care for HIV, viral hepatitis, tuberculosis, and sexual transmitted infections in Europe
